# 3-(4-Methoxy­phen­yl)-1-(2-nitrophen­yl)prop-2-en-1-one

**DOI:** 10.1107/S1600536809048120

**Published:** 2009-11-18

**Authors:** Huan-Mei Guo, Le-Qing Liu, Jie Yang, Fang-Fang Jian

**Affiliations:** a Microscale Science Institute , Weifang University, Weifang 261061, People’s Republic of China; b the Seventh Middle School of Wei Fang, Weifang 261041, People’s Republic of China

## Abstract

The title compound, C_16_H_13_NO_4_, was prepared from 2-nitrylhypnone [systematic name: 1-(2-nitrophenyl)ethanone] and 4-methoxy­benzophenone by a Claisen–Schmidt condensation. The dihedral angle formed by the two benzene rings is 80.73 (2). The crystal packing is stabilized by inter­molecular C—H⋯O hydrogen bonds.

## Related literature

For the biological activity of chalcones, see: Anto *et al.* (1994 ▶); De Vincenzo *et al.* (2000 ▶); Dimmock *et al.* (1998 ▶); Hsieh *et al.* (1998 ▶). For a related structure, see: Fun *et al.* (2008 ▶).
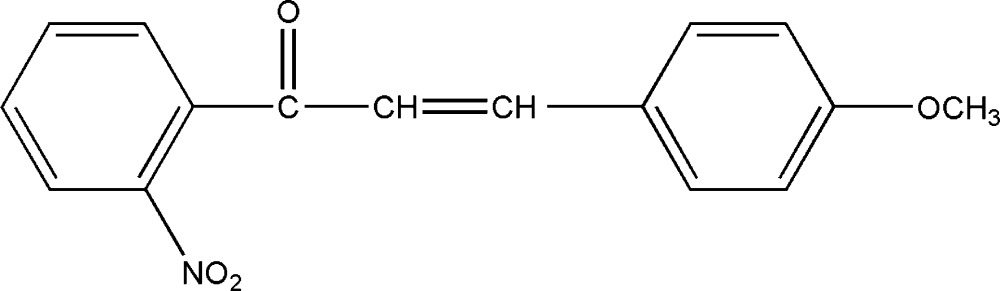


## Experimental

### 

#### Crystal data

C_16_H_13_NO_4_
*M* *_r_* = 283.27
Monoclinic, 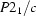

*a* = 11.594 (2) Å
*b* = 7.7736 (16) Å
*c* = 15.174 (3) Å
β = 94.59 (3)°
*V* = 1363.1 (5) Å^3^
*Z* = 4
Mo *K*α radiation
μ = 0.10 mm^−1^
*T* = 293 K
0.21 × 0.18 × 0.10 mm

#### Data collection

Bruker SMART CCD area-detector diffractometer
Absorption correction: none
12773 measured reflections
3107 independent reflections
2667 reflections with *I* > 2σ(*I*)
*R*_int_ = 0.018

#### Refinement

*R*[*F* ^2^ > 2σ(*F* ^2^)] = 0.041
*wR*(*F* ^2^) = 0.124
*S* = 1.08
3107 reflections
190 parameters
H-atom parameters constrained
Δρ_max_ = 0.19 e Å^−3^
Δρ_min_ = −0.22 e Å^−3^

### 

Data collection: *SMART* (Bruker, 1997 ▶); cell refinement: *SAINT* (Bruker, 1997 ▶); data reduction: *SAINT*; program(s) used to solve structure: *SHELXS97* (Sheldrick, 2008 ▶); program(s) used to refine structure: *SHELXL97* (Sheldrick, 2008 ▶); molecular graphics: *SHELXTL* (Sheldrick, 2008 ▶); software used to prepare material for publication: *SHELXTL*.

## Supplementary Material

Crystal structure: contains datablocks global, I. DOI: 10.1107/S1600536809048120/fl2279sup1.cif

Structure factors: contains datablocks I. DOI: 10.1107/S1600536809048120/fl2279Isup2.hkl

Additional supplementary materials:  crystallographic information; 3D view; checkCIF report

## Figures and Tables

**Table 1:** Hydrogen-bond geometry (Å, °)

*D*—H⋯*A*	*D*—H	H⋯*A*	*D*⋯*A*	*D*—H⋯*A*
C16—H16*A*⋯O1^i^	0.93	2.51	3.249 (1)	136
C14—H14*A*⋯O3^ii^	0.93	2.59	3.259 (2)	129

Symmetry codes: (i) 
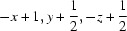
; (ii) 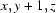.

**Fig. 1.**
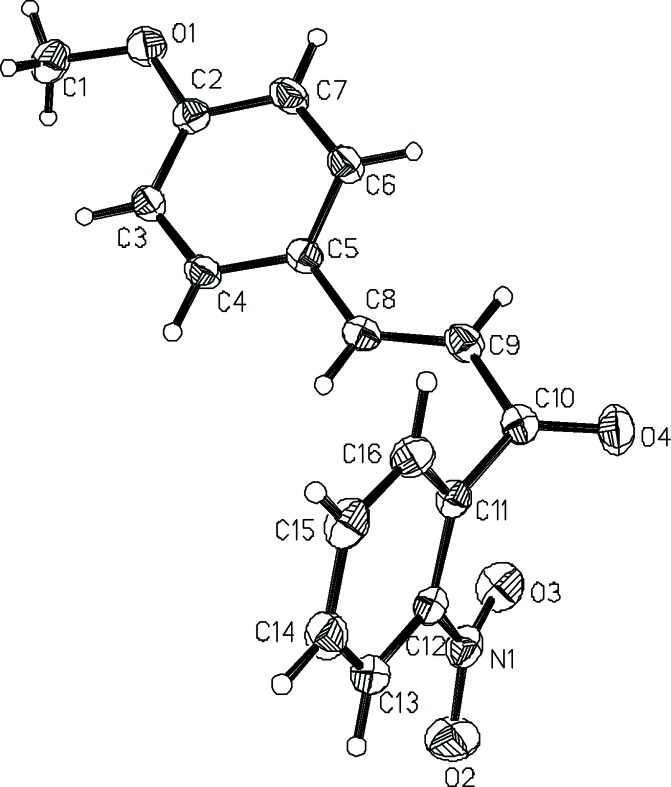
The molecular structure of the title compound with the atom-labeling scheme. Displacement ellipsoids are drawn at the 30% probability level.

**Fig. 2.**
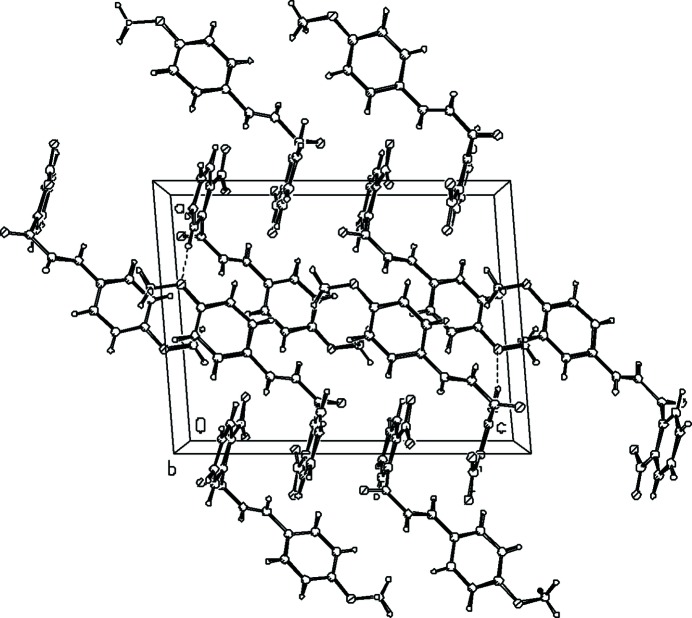
The crystal packing of the title compound,viewed along a axis.

## supplementary crystallographic information

### Comment

Among flavonoids, chalcones have been identified as interesting compounds having multiple biological actions which include antiinflammatory (Hsieh *et al.*,1998)and antioxidant (Anto *et al.*,1994). Of particular interest, the effectiveness of chalcones againest cancer has been investigated (De Vincenzo *et al.*, 2000; Dimmock *et al.*,1998). As part of our search for new biologically active compounds we synthesized the title compound(I) and report its crystal structure herein.

Scheme I

The molecule (I) (Fig. 1) is made up of two essentially planar segments. The atoms O1, C1,C2, C3, C4, C5, C6 and C7 make up one segment (largest deviation being 0.018Å for C6) with the nitro phenyl group being the second planar segment (largest deviation 0.0177Å for N1).The dihedral angel formed by the two planes is 81.07 (2)°. All of the bond lengths and bond angles are in normal ranges and comparable to those found in a related structure (Fun *et al.*, 2008). In the crystal structure, the molecules are stacked along the *b* axis and linked *via* C—H···O interactions (Fig. 2).

### Experimental

The title compound(I) was prepared by the process as following: A mixture of the 2-nitrylhypnone (0.02 mol), 4-methoxybenzophenone(0.02 mol) and 10%NaOH(10 ml) was stirred in ethanol(30 ml) for 3 h to afford the title compound(yield78%).Single crystals suitable for X-ray measurements were obtained by recrystallization from ethyl acetate at room temperature.

### Refinement

H atoms were positioned geometrically and allowed to ride on their parent atoms, with C—H distances of0.93–0.96 Å, and with *U*_iso_(H) = 1.2*U*_eq_ of the parent atoms.

### Crystal data

**Table:**

C_16_H_13_NO_4_	*F*(000) = 592
*M**_r_* = 283.27	*D*_x_ = 1.380 Mg m^−^^3^
Monoclinic, *P*2_1_/*c*	Mo *K*α radiation, λ = 0.71073 Å
Hall symbol: -P 2ybc	Cell parameters from 2667 reflections
*a* = 11.594 (2) Å	θ = 3.1–27.5°
*b* = 7.7736 (16) Å	µ = 0.10 mm^−^^1^
*c* = 15.174 (3) Å	*T* = 293 K
β = 94.59 (3)°	Bar, yellow
*V* = 1363.1 (5) Å^3^	0.21 × 0.18 × 0.10 mm
*Z* = 4	

### Data collection

**Table:**

Bruker sMART CCD area-detector diffractometer	2667 reflections with *I* > 2σ(*I*)
Radiation source: fine-focus sealed tube	*R*_int_ = 0.018
graphite	θ_max_ = 27.5°, θ_min_ = 3.1°
phi and ω scans	*h* = −15→15
12773 measured reflections	*k* = −10→8
3107 independent reflections	*l* = −19→19

### Refinement

**Table:**

Refinement on *F*^2^	Primary atom site location: structure-invariant direct methods
Least-squares matrix: full	Secondary atom site location: difference Fourier map
*R*[*F*^2^ > 2σ(*F*^2^)] = 0.041	Hydrogen site location: inferred from neighbouring sites
*wR*(*F*^2^) = 0.124	H-atom parameters constrained
*S* = 1.08	*w* = 1/[σ^2^(*F*_o_^2^) + (0.0747*P*)^2^ + 0.157*P*] where *P* = (*F*_o_^2^ + 2*F*_c_^2^)/3
3107 reflections	(Δ/σ)_max_ < 0.001
190 parameters	Δρ_max_ = 0.19 e Å^−^^3^
0 restraints	Δρ_min_ = −0.22 e Å^−^^3^

### Special details

**Table:**

Geometry. All e.s.d.'s (except the e.s.d. in the dihedral angle between two l.s. planes) are estimated using the full covariance matrix. The cell e.s.d.'s are taken into account individually in the estimation of e.s.d.'s in distances, angles and torsion angles; correlations between e.s.d.'s in cell parameters are only used when they are defined by crystal symmetry. An approximate (isotropic) treatment of cell e.s.d.'s is used for estimating e.s.d.'s involving l.s. planes.
Refinement. Refinement of *F*^2^ against ALL reflections. The weighted *R*-factor *wR* and goodness of fit *S* are based on *F*^2^, conventional *R*-factors *R* are based on *F*, with *F* set to zero for negative *F*^2^. The threshold expression of *F*^2^ > σ(*F*^2^) is used only for calculating *R*-factors(gt) *etc*. and is not relevant to the choice of reflections for refinement. *R*-factors based on *F*^2^ are statistically about twice as large as those based on *F*, and *R*- factors based on ALL data will be even larger.

### Fractional atomic coordinates and isotropic or equivalent isotropic displacement parameters (Å^2^)

**Table:**

	*x*	*y*	*z*	*U*_iso_*/*U*_eq_	
C8	0.72395 (9)	0.18277 (14)	0.22201 (7)	0.0385 (2)	
H8A	0.7810	0.2655	0.2340	0.046*	
O4	0.82958 (9)	0.02414 (13)	0.02294 (6)	0.0603 (3)	
C5	0.63411 (9)	0.17369 (13)	0.28408 (7)	0.0371 (2)	
N1	1.07316 (9)	0.10395 (13)	0.16402 (6)	0.0447 (2)	
C2	0.46388 (9)	0.17332 (14)	0.40548 (8)	0.0402 (3)	
C7	0.44279 (10)	0.09466 (16)	0.32265 (8)	0.0482 (3)	
H7A	0.3714	0.0436	0.3073	0.058*	
O3	1.01265 (9)	−0.02297 (12)	0.16921 (7)	0.0611 (3)	
C16	0.85299 (10)	0.42736 (15)	0.08146 (8)	0.0442 (3)	
H16A	0.7755	0.4334	0.0604	0.053*	
C11	0.90152 (9)	0.26829 (14)	0.10358 (7)	0.0359 (2)	
C12	1.01784 (9)	0.26616 (13)	0.13472 (7)	0.0362 (2)	
C6	0.52710 (10)	0.09263 (15)	0.26394 (8)	0.0445 (3)	
H6A	0.5130	0.0366	0.2100	0.053*	
C13	1.08378 (10)	0.41407 (16)	0.14300 (8)	0.0454 (3)	
H13A	1.1615	0.4087	0.1636	0.054*	
C9	0.73401 (10)	0.08635 (15)	0.15007 (8)	0.0435 (3)	
H9A	0.6811	−0.0023	0.1384	0.052*	
C14	1.03253 (12)	0.57038 (15)	0.12026 (9)	0.0514 (3)	
H14A	1.0760	0.6710	0.1254	0.062*	
C4	0.65192 (9)	0.25538 (14)	0.36590 (8)	0.0414 (3)	
H4A	0.7213	0.3129	0.3799	0.050*	
O2	1.17769 (9)	0.10328 (15)	0.18354 (8)	0.0719 (3)	
C10	0.82364 (10)	0.11242 (15)	0.08857 (7)	0.0409 (3)	
C15	0.91755 (12)	0.57743 (15)	0.09011 (9)	0.0493 (3)	
H15A	0.8831	0.6828	0.0755	0.059*	
C3	0.56915 (10)	0.25320 (14)	0.42687 (8)	0.0410 (3)	
H3A	0.5842	0.3051	0.4818	0.049*	
O1	0.37527 (8)	0.16582 (14)	0.45895 (6)	0.0582 (3)	
C1	0.39013 (13)	0.24330 (19)	0.54405 (9)	0.0596 (4)	
H1A	0.3210	0.2283	0.5740	0.089*	
H1B	0.4054	0.3639	0.5378	0.089*	
H1C	0.4540	0.1900	0.5778	0.089*	

### Atomic displacement parameters (Å^2^)

**Table:**

	*U*^11^	*U*^22^	*U*^33^	*U*^12^	*U*^13^	*U*^23^
C8	0.0357 (5)	0.0373 (5)	0.0419 (5)	−0.0036 (4)	−0.0006 (4)	0.0031 (4)
O4	0.0715 (6)	0.0603 (6)	0.0498 (5)	−0.0125 (5)	0.0103 (4)	−0.0185 (4)
C5	0.0369 (5)	0.0338 (5)	0.0400 (5)	−0.0028 (4)	0.0002 (4)	0.0032 (4)
N1	0.0496 (5)	0.0450 (5)	0.0397 (5)	0.0120 (4)	0.0042 (4)	−0.0005 (4)
C2	0.0387 (5)	0.0357 (5)	0.0467 (6)	−0.0029 (4)	0.0060 (4)	0.0029 (4)
C7	0.0404 (6)	0.0522 (7)	0.0516 (7)	−0.0158 (5)	0.0010 (5)	−0.0042 (5)
O3	0.0728 (6)	0.0398 (5)	0.0711 (6)	0.0075 (4)	0.0090 (5)	0.0099 (4)
C16	0.0426 (6)	0.0422 (6)	0.0481 (6)	0.0065 (4)	0.0056 (5)	0.0045 (5)
C11	0.0386 (5)	0.0361 (5)	0.0338 (5)	0.0004 (4)	0.0068 (4)	−0.0003 (4)
C12	0.0396 (5)	0.0365 (5)	0.0330 (5)	0.0047 (4)	0.0061 (4)	−0.0024 (4)
C6	0.0438 (6)	0.0472 (6)	0.0419 (6)	−0.0112 (5)	−0.0004 (5)	−0.0041 (5)
C13	0.0394 (5)	0.0485 (7)	0.0487 (6)	−0.0032 (5)	0.0060 (5)	−0.0088 (5)
C9	0.0432 (6)	0.0406 (6)	0.0466 (6)	−0.0093 (4)	0.0018 (5)	−0.0011 (5)
C14	0.0590 (7)	0.0378 (6)	0.0590 (7)	−0.0104 (5)	0.0145 (6)	−0.0077 (5)
C4	0.0349 (5)	0.0418 (6)	0.0467 (6)	−0.0067 (4)	−0.0012 (4)	−0.0029 (5)
O2	0.0501 (6)	0.0756 (7)	0.0877 (8)	0.0208 (5)	−0.0089 (5)	0.0045 (6)
C10	0.0440 (6)	0.0387 (6)	0.0396 (5)	−0.0008 (4)	0.0007 (4)	−0.0014 (4)
C15	0.0618 (7)	0.0337 (6)	0.0537 (7)	0.0077 (5)	0.0134 (6)	0.0041 (5)
C3	0.0406 (6)	0.0400 (6)	0.0417 (5)	−0.0026 (4)	−0.0006 (4)	−0.0044 (4)
O1	0.0492 (5)	0.0682 (6)	0.0593 (5)	−0.0160 (4)	0.0177 (4)	−0.0100 (5)
C1	0.0642 (8)	0.0620 (8)	0.0548 (7)	0.0018 (6)	0.0188 (6)	−0.0020 (6)

### Geometric parameters (Å, °)

**Table:**

C8—C9	1.3369 (17)	C11—C10	1.5174 (15)
C8—C5	1.4609 (16)	C12—C13	1.3809 (16)
C8—H8A	0.9300	C6—H6A	0.9300
O4—C10	1.2159 (15)	C13—C14	1.3841 (18)
C5—C4	1.3951 (16)	C13—H13A	0.9300
C5—C6	1.4031 (15)	C9—C10	1.4653 (17)
N1—O3	1.2168 (14)	C9—H9A	0.9300
N1—O2	1.2242 (15)	C14—C15	1.3753 (19)
N1—C12	1.4674 (14)	C14—H14A	0.9300
C2—O1	1.3604 (14)	C4—C3	1.3854 (17)
C2—C3	1.3846 (16)	C4—H4A	0.9300
C2—C7	1.4015 (17)	C15—H15A	0.9300
C7—C6	1.3743 (18)	C3—H3A	0.9300
C7—H7A	0.9300	O1—C1	1.4227 (17)
C16—C15	1.3866 (18)	C1—H1A	0.9600
C16—C11	1.3885 (15)	C1—H1B	0.9600
C16—H16A	0.9300	C1—H1C	0.9600
C11—C12	1.3929 (15)		
			
C9—C8—C5	127.78 (10)	C12—C13—H13A	120.5
C9—C8—H8A	116.1	C14—C13—H13A	120.5
C5—C8—H8A	116.1	C8—C9—C10	123.72 (10)
C4—C5—C6	117.62 (10)	C8—C9—H9A	118.1
C4—C5—C8	119.30 (9)	C10—C9—H9A	118.1
C6—C5—C8	123.02 (10)	C15—C14—C13	120.22 (11)
O3—N1—O2	123.06 (11)	C15—C14—H14A	119.9
O3—N1—C12	118.43 (10)	C13—C14—H14A	119.9
O2—N1—C12	118.50 (11)	C3—C4—C5	121.84 (10)
O1—C2—C3	124.96 (11)	C3—C4—H4A	119.1
O1—C2—C7	115.49 (10)	C5—C4—H4A	119.1
C3—C2—C7	119.55 (11)	O4—C10—C9	122.22 (11)
C6—C7—C2	120.28 (10)	O4—C10—C11	120.10 (11)
C6—C7—H7A	119.9	C9—C10—C11	117.30 (9)
C2—C7—H7A	119.9	C14—C15—C16	119.96 (11)
C15—C16—C11	121.41 (11)	C14—C15—H15A	120.0
C15—C16—H16A	119.3	C16—C15—H15A	120.0
C11—C16—H16A	119.3	C2—C3—C4	119.58 (10)
C16—C11—C12	117.07 (10)	C2—C3—H3A	120.2
C16—C11—C10	116.75 (10)	C4—C3—H3A	120.2
C12—C11—C10	126.14 (9)	C2—O1—C1	118.75 (10)
C13—C12—C11	122.33 (10)	O1—C1—H1A	109.5
C13—C12—N1	117.55 (10)	O1—C1—H1B	109.5
C11—C12—N1	120.05 (10)	H1A—C1—H1B	109.5
C7—C6—C5	121.06 (11)	O1—C1—H1C	109.5
C7—C6—H6A	119.5	H1A—C1—H1C	109.5
C5—C6—H6A	119.5	H1B—C1—H1C	109.5
C12—C13—C14	119.00 (11)		

### Hydrogen-bond geometry (Å, °)

**Table:**

*D*—H···*A*	*D*—H	H···*A*	*D*···*A*	*D*—H···*A*
C16—H16A···O1^i^	0.93	2.51	3.249 (1)	136
C14—H14A···O3^ii^	0.93	2.59	3.259 (2)	129

Symmetry codes: (i) −*x*+1, *y*+1/2, −*z*+1/2; (ii) *x*, *y*+1, *z*.
